# Patch Test Preparations: Basis and State-of-the-Art Modern Diagnostic Tools for Contact Allergy

**DOI:** 10.3390/jcm14217521

**Published:** 2025-10-23

**Authors:** Julia Zimmer, Sonja Neimanis, Sandra Schmidt, Steffen Schubert, Vera Mahler

**Affiliations:** 1 Allergology Division, Paul-Ehrlich-Institut, 63225 Langen, Germany; 2 Information Network of Departments of Dermatology (IVDK), Institute at the University Medical Centre Göttingen, 37073 Göttingen, Germany

**Keywords:** epicutaneous patch test preparations, allergic contact dermatitis, contact allergy, marketing authorization, quality defects, test allergen, Type IV-allergy, non-authorized patch test products

## Abstract

Reliable quality of epicutaneous patch test (PT) preparations is a prerequisite for establishing a robust diagnosis in patients with suspected allergic contact dermatitis due to delayed-type sensitization. It is difficult to identify potential quality issues in daily practice, since confirmatory methods are lacking and assessment of PT-relevance is predominantly based on patients’ history and exposure. The quality of PT products can be affected, e.g., by the properties of the active substance, an insufficient development of the PT preparation or issues during manufacturing. Resulting quality deficiencies can cause both false-negative and false-positive test results. As PT preparations are medicinal products according to Directive 2001/83/EC, they require a marketing authorization (MA) entailing assessment of quality, safety and efficacy by the competent authorities. The corresponding product dossier is the basis for MA. It is continuously updated, e.g., upon change of a source material supplier, ensuring comparability of the respective product over time. Compliance with regulatory requirements is a crucial foundation for sustainable quality to prevent product deficiencies, ensuring reliable test results in practice. Harmonization across the EU is important to ensure the widespread availability of high-quality PT products. This review presents the MA requirements of PT preparations in the EU, as well as challenges previously reported by physicians.

## 1. Introduction

Allergen products for *in vivo* diagnosis can be separated into two major groups: Extract-based products prepared from allergenic source material like pollen for diagnosis of immediate-type hypersensitivity (Type I allergy, IgE-mediated) and epicutaneous patch test (PT) preparations for diagnosis of delayed-type hypersensitivity (Type IV allergy, cell-mediated) in patients with suspected allergic contact dermatitis. While Type I allergy is typically caused by allergenic proteins, Type IV allergy is triggered by small molecules (haptens), which can only elicit an immune response after binding to a larger carrier molecule (i.e., protein haptenization) [1]. Identification of the causative allergen is challenging, first of all, because several thousand substances have been identified as potential contact allergens [2]. In addition, the interpretation of PT results is typically limited to comparing the test outcome with the patient’s medical history and identifying relevant sources of exposure, as there are no alternative or confirmatory tests for most haptens. One exception is the so-called lymphocyte transformation test (LTT), but this method has so far only been validated for a minimal number of haptens [3]. Similarly, the activation-induced marker (AIM) assay is currently being developed; the first results were presented for nickel only [4]. Without a validated positive control or confirmatory test, detecting potential quality issues in PT preparations can be challenging in everyday clinical practice. In addition, these specific problems are unfortunately often not reported or discussed in the literature.

Compared to other medicinal products, the composition of epicutaneous PT preparations is relatively simple because the active substance is usually directly mixed into either petrolatum, water or further solvents like ethanol in rare cases. Nevertheless, the quality of the allergen preparation used in PTs can be compromised by a variety of factors, such as specific properties of the active substance, improper formulation of the test preparation, or issues that arise during manufacturing. These issues can lead to problems like reduced stability (e.g., due to chemical degradation), incorrect concentrations of the active ingredient, polymerization, particle formation, unintended contamination with other compounds, impurities, or unexpected reactions between hapten with either the matrix, the container or the test chamber. These quality defects will be addressed during the assessment of the marketing authorization (MA) dossier. However, in non-authorized PT products, unfortunately, many potential quality deficiencies are likely to remain undetected by the physician. Hence, it can be challenging to avoid false diagnoses in cases where quality deficiencies result in either invalid, false-negative or false-positive reactions. Ultimately, any false diagnosis can significantly impact the patients’ quality of life, e.g., if patients avoid a substance they are actually not sensitized to.

Although regulatory practices for this category of products are heterogeneous in different Member States (MS) at the time given due to various exemptions in the European Union (EU) [4,5], epicutaneous PT preparations are clearly classified as *in vivo* diagnostics. They are thus medicinal products under Directive 2001/83/EC (the Community Code on Medicinal Products for Human Use). This means that all such products that are either prepared industrially or manufactured by a method involving an industrial process must undergo a formal MA process before they are placed on the market. This includes evaluation of their quality, safety, and efficacy by the competent authority in charge. In Germany, the Paul-Ehrlich-Institut is responsible for all procedures dealing with *in vivo* test allergens. The product dossier follows the general requirements laid down in Directive 2001/83/EC and serves as the foundation for this approval. So far, most authorized PT products have obtained national MAs in one MS; a few have obtained MA in several EU MS via a Mutual recognition procedure (MRP) or a Decentralized procedure (DCP). In Germany, currently 167 PT preparations with a MA are marketable (https://www.pei.de/EN/medicinal-products/allergens/epicutaneous-test/epicutaneous-test-node.html, last accessed on 6 October 2025). The most common regulatory pathway for epicutaneous PT preparations in Germany is the well-established use of MA according to Article 10a (Table 1), which is based on bibliographic clinical data only (i.e., no own clinical studies). However, other legal bases for MA, such as a full MA (according to Article 8 (3)), can be selected depending on the available data set (Table 1). Importantly, the content of the quality module of the dossier is similar for all possible types of MA and is regularly updated, also after MA has been granted. Such an update is necessary, for example, if there is a change in the supplies of the raw material. This ensures consistency and batch-to-batch comparability of the product over time. Compliance with these regulatory standards is crucial for maintaining product quality, avoiding defects, and ensuring the reliability of test results in clinical settings.

PT-substances historically have been distributed as medicinal products without an MA in many MS applying Article 5 of Directive 2001/83/EC, allowing for exemptions from the above-mentioned MA requirement under special conditions [6]:

“A MS may […] to fulfil special needs, exclude from the provisions of this Directive medicinal products […] formulated in accordance with the specifications of an authorized health-care professional and for use by his individual patients on his direct personal responsibility.” This exemption has been applied differently in several MS and led to a heterogenous authorization status of PT products [6]: In some MS, PT-substances have been distributed without MA under transitional provisions, on exceptional rules or as named patient products (NPP), although according to Article 5 of the Directive 2001/83/EC, by definition, the directive does not apply to multidose diagnostics (e.g., a test allergen preparation in a container used for more than one diagnostic test). PT preparations without MA are not independently assessed for quality, safety and efficacy. To harmonize national approaches on the regulation of allergen products and thereby strengthen the availability of high-quality products across the EU, a guideline was developed that contains differentiated recommendations on the regulatory procedures to be applied for diagnostics (specifically PT preparations), allergen immunotherapy products or NPPs [7].

**Figure 1:**
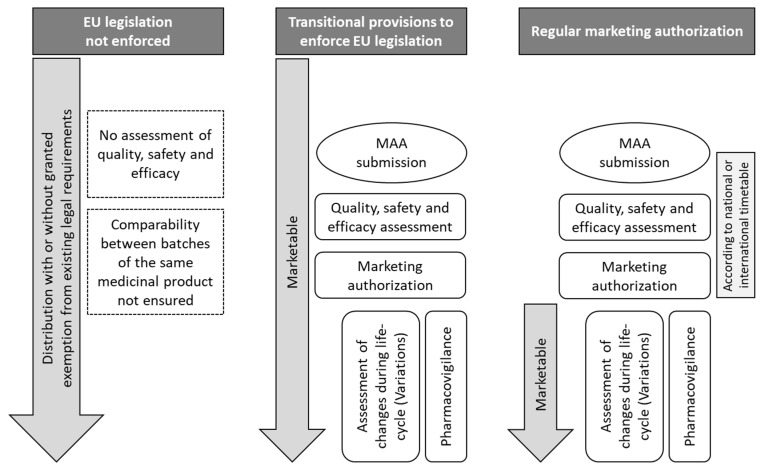
Regulatory status and processes of epicutaneous patch test preparations in the EU. In the majority of MS, only PT preparations without MA are available. These are not assessed by the authorities for their quality, safety and efficacy. Few MS have implemented transitional provisions enforcing the requirement for MA for PT preparations, which have already been on the market. After submission of the MAA by the pharmaceutical company, the competent authority assesses the quality, safety and efficacy of the PT preparations. However, in contrast to the regular MA process, under transitional provisions, PT preparations are marketable in the respective MS before the MA is granted. In addition, the duration of the MA procedure under transitional provisions may be considerably longer, e.g., because the authorities face the parallel submission of numerous MAA. MA: Marketing authorization, MAA: Marketing authorization application, PT: Patch test.

Accordingly, several EU countries have in recent years enforced the requirement for MA for epicutaneous PT preparations. In Spain, a notification period for all allergen products on the Spanish market ended in January 2024. In total, 2807 diagnostic allergen products for human use have been notified, including 1431 haptens (personal communication M. Timon, Agencia Española de Medicamentos y Productos Sanitarios). For these products, a marketing authorization application (MAA) must be filed by 31 December 2030. Until this deadline, these products remain marketable. In Italy, the situation is more complex [8]. Based on a Ministerial Decree dated 13 December 1991, epicutaneous PT preparations which have been marketed until this date could be notified and remain marketable ope legis until a decision is reached on MA. In 2017, Determina DG2130/2017 reactivated these authorization processes and currently, products one after another undergo MA. In addition, Determina DG 98/2021, DG 98/2022 and DG 98/2023 enabled marketability of more than 200 additional epicutaneous PT preparations, which were not covered by the ministerial decree in 1991. For these products, MAA will need to be filed by the end of 2026; otherwise, the marketability will expire. Notably, the large number of products currently marketable both in Italy and Spain has so far not undergone assessment of quality, safety and efficacy, which will take place during the respective MA procedures (Figure 1). While these activities are currently limited to two MS and are still in progress, the number of authorized PT preparations remains very low in the EU. In the majority of MS, no authorized PT preparations are available.

Overall, the widespread use of unauthorized PT products can not only be traced back to the undeniable lack of availability, but also to a knowledge gap regarding the benefits of authorized PT preparations. Consequences of potential quality defects in PT preparations are difficult to identify and thus underreported. This review aims to fill this knowledge gap by outlining the regulatory requirements for MA of epicutaneous PT preparations in conjunction with challenges reported by clinicians. Thus, this review highlights the benefits of using authorized diagnostic products as well as the related need for harmonization of the regulatory landscape in Europe, which to date still appears inhomogeneous for PT preparations due to differently applied exemptions (Art. 5, Directive 2001/83/EC).

## 2. Challenges Observed in the Clinic

Despite its long-time use in clinical practice for 120 years, patch testing remains a challenging task. The first challenge is that not all culprit substances have commercial PT preparations available. Thus, not all possible diagnostic problems can be solved with commercial haptens, and testing with patients’ own products or self-produced preparations is necessary [9]. The European Society of Contact Dermatitis guideline for diagnostic patch testing gives valuable recommendations for a hands-on approach to prepare PT preparations from the patient’s own materials [9], which are evidently less standardized than commercial PT-substances.

**Figure 2:**
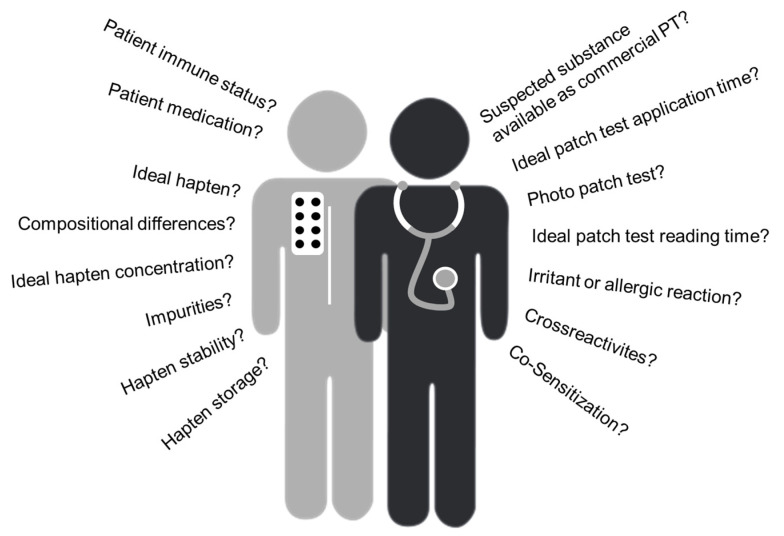
Potential challenges when performing epicutaneous patch testing. Clinicians face a number of questions when performing patch testing, ranging from general to patient- and hapten-specific considerations.

However, even when using commercial PT preparations, the outcome of a patch test can be influenced by a multitude of factors which need to be taken into account by the physician (Figure 2). Such factors can be the cause of false-positive and false-negative results and include patient selection, patient immune status and medication like immunosuppressive therapy, patch test reading time and handling, including application of excessive or insufficient doses, as well as exposure to ultraviolet light [10]. In addition, many Type IV allergens are also irritants, which can make it challenging to differentiate between irritant and allergic reactions. Common examples of such problematic PT preparations are hydroperoxides, sodium benzoate and glucosides [11,12,13]. Furthermore, simultaneous testing of the same allergen, for example, on the right and the left arm or in different areas of the back, revealed variability in test results depending on the tested hapten [14], adding to patch test result uncertainty.

In addition, the selection of the most suitable test concentration of a PT preparation is crucial. As a consequence, the German Contact Dermatitis Research Group (DKG) regularly critically revalidates its test series recommendations based on new evidence (including parallel patch testing of different concentrations and changing exposures), e.g., for methyldibromoglutaronitrile (MDBGN) [15]. Its current test recommendation is MDBGN 0.2% pet (https://dkg.ivdk.org/testreihen.html; last accessed on 27 August 2025). Increasing the PT concentration of MDBGN 0.2% pet. to 0.3% pet. in 2016 due to temporary supply constraints has caused a sudden rise in positive patch test reactions from 2.0% to more than 4.5%, although there is no longer any widespread exposure to MDBGN since 2008; the new, higher test concentration elicits many false-positive test reactions and explains the sudden rise in positive patch test reactions since 2016 [16].

Also, the selection of the correct active substance can be challenging, as was shown for the common fragrances linalool and limonene [17,18,19,20]: It was demonstrated that testing with hydroperoxides of the respective substances yields a much higher rate of positive reactions compared to non-oxidized substances, but with the drawback of more doubtful/irritant reactions and uncertainty regarding the clinical relevance. In addition, standardization of such active substances and their oxidation products is challenging.

Finally, hapten-specific characteristics have to be considered. For example, it was shown for p-toluene diamine (PTD) preparations that the amount of PTD contained in the PT preparation decreased rapidly over time, but patch test reactions were not altered, because reaction products of PTD are additional important allergens to which most allergic patients are co-sensitized [21]. However, for the vast majority of haptens, degradation of the active substance in the PT preparation is likely to result in false-negative results, as seen, for example, for the 4-component mercapto mix, in which the mercaptobenzothiazole (MBT) component was shown to degrade [22,23]. Pre-loading the test chambers with PT preparations a long time in advance of application can result in false-negative reactions in case of volatile haptens like fragrances [24].

Another example of active substance-specific pitfalls in patch testing has been recently described for propolis, which is part of the baseline series in Germany [25]. In contrast to propolis harvested in China, Brazilian propolis (no MA in Germany) caused an unusually high number of positive reactions of unclear clinical relevance [26]. It could be shown that the difference was possibly due to bacterial contamination and/or impurities contained in the Brazilian propolis [25]. This example also highlights the general challenges that can be associated with changes in active substance suppliers. Especially in the case of natural materials, such as essential oil products marketed under the same name, might exhibit very different compositions, also regarding allergenic constituents [27,28,29].

Mose et al. compared the hapten content of PT preparations of methyl methacrylate (MMA) and 2-hydroxyethyl methacrylate (2-HEMA) from different suppliers to the amount labelled, observing lower hapten content in all tested syringes containing MMA and in 1/3 of the tested syringes containing 2-HEMA. Apart from this lack of stability, a lack of homogeneity within the syringe was detected in multiple samples for MMA, with lower MMA values at the tip of the syringe compared to the segment near the piston [30]. Similar results have been collected for a number of PT preparations, especially in the case of volatile active substances like glutaraldehyde and formaldehyde [31].

Another problem associated with the nature of certain contact allergens is impurities. The problem has been exemplarily described for PT preparations containing acrylates and methacrylates. Analysis via gas chromatography and mass spectrometry revealed purities of certain (meth)acrylates of only 81% [32]. Another product group prone to impurities is disperse dyes, which are known to commonly contain a significant amount of impurities. Several textile dyes analyzed by Ryberg et al. showed lower and varying concentrations compared to the PT preparation label, varying impurities, including contamination with other textile dyes, as well as, in some cases, even absence of the labelled hapten [33]. Similar observations were described for a mix of disperse blue dyes 106 and 124, whereupon the authors concluded that quality control ensuring accurate test concentrations of haptens is the prerequisite for valid diagnosis of contact allergy and comparable epidemiological data [34]. It was also recently reported that several textile dyes contained in a PT mix had their product labelling corrected by the manufacturer when it became apparent that the actual dye content was substantially lower than assumed [35].

In view of the various ways PT preparation characteristics and quality can influence patch test results, it is not surprising that the S3 Guideline “Epicutaneous patch testing with contact allergens and drugs” strongly recommends using authorized PT preparations whenever possible [36], since the above issues will be dealt within the MAA process.

It should also be noted that companies marketing authorized products within the EU are obliged to have pharmacovigilance systems in place and bear liability for the effects of their products used under the terms of the marketing authorization. Prescribers and dispensers should be obliged to report all adverse events to the appropriate authorities. However, with “special medicines” and genuine unlicensed medicines, there is no such obligation for manufacturers to provide pharmacovigilance because all product liability rests with the prescriber and the multidisciplinary team [37].

## 3. Specific Regulatory Practice for Patch Tests

First of all, it should be highlighted that regulatory requirements for epicutaneous PT preparations for the diagnosis of Type IV allergy differ from extract-based allergen products for the diagnosis and therapy of Type I allergy. The latter are mainly controlled according to the Ph. Eur. Monograph on Allergen Products [38] and the Guideline on Allergen Products: Production and Quality Issues [39]. These, however, exclude from their scope chemicals as active ingredients which are used for the diagnosis of contact dermatitis. Nevertheless, the Ph. Eur., as well as other general guidelines like the ICH guidelines of the International Council for Harmonisation of Technical Requirements for Pharmaceuticals for Human Use (ICH) (e.g., ICH Q2 (R1), ICH Q5, ICH Q6B), play a central role in quality control of epicutaneous PT preparations. The main regulatory documents applicable to allergen products for *in vivo* diagnosis of type IV allergy have recently been summarized in Zimmer&Mahler, 2024 [5]. Apart from the central role of Directive 2001/83/EC in defining products used for *in vivo* diagnosis of allergies as medicinal products, it also defines the extent of data to be submitted for MAA in Annex I (Table 1).

With regard to quality, control of the finished product is especially important in epicutaneous PT preparations because the utilized active substances are commonly not of Ph. Eur. quality but “atypical” active substances in the field of medicinal products. They encompass dyes, adhesives, metals, preservatives, fragrances, rubber chemicals and many other substance classes. These substances are usually not produced under GMP conditions or with the intention of being included in a medicinal product. Consequently, quality control by the respective manufacturers and suppliers is, in some cases, limited to a minimum. Therefore, regulatory requirements have been adapted, and GMP requirements only apply after the material has reached the manufacturer of the epicutaneous PT preparation (Table 2). Though unavoidable due to the nature of the active substances, this practice makes unambiguous verification of identity, homogeneity and active substance content of the finished product all the more important. Control of active substance identity can be especially challenging due to the broad portfolio required for meaningful diagnosis, which often encompasses chemically related substances like thiram and disulfiram. At the same time, verification of identity is highly important to ensure that the active substance is indeed the intended one and has not, e.g., been interchanged by mistake by the supplier or during PT production.

Apart from the waiver of GMP requirements for the active substance, some other deviations from common regulatory requirements are acceptable due to the specific nature of PT preparation (Table 2). For example, the recently published *Guideline on Allergen Product Development for Immunotherapy and Allergy Diagnosis in Moderate to Low-Sized Study Populations* [40] allows a matrix approach for process validation, in deviation from the Guideline on process validation for finished products [41]. This matrix approach can limit the need for process validation data to a justified set of representative products, provided that all products in the respective validation group share an identical manufacturing process and are similar in concentration, batch size and dosage form.

As described, some regulatory rules applicable to medicinal products are simplified for epicutaneous PT preparations. However, other central requirements like the need for validated methods for quality control, control of microbiological quality or the necessity to demonstrate stability for each hapten over the assigned shelf life, do not deviate from those of other medicinal products, ensuring batch-to-batch consistency.

Importantly, authorization of epicutaneous PT preparations by national competent authorities does not only encompass assessment of quality, but also assessment of pre-clinical and clinical data (Table 2). As the majority of active substances in PT preparations are commonly used in everyday products and occupational settings, non-clinical data is usually available to a certain degree and can be provided, e.g., in the form of technical data sheets. The new *Guideline on Allergen Product Development for Immunotherapy and Allergy Diagnosis in Moderate to Low-Sized Study Population* [40] provides guiding principles on the extent and compilation of such already existing data. Likewise, the guideline offers guidance on clinical data, as the *EMA Guideline on clinical evaluation of diagnostic agents* [42] can usually not be applied to PT preparations due to the absence of a standard of truth and thus the impossibility of determining sensitivity and specificity. Alternative parameters like positivity ratio (PR) and reaction index (RI) [43,44] obtained in large register studies reflecting on the performance of a PT preparation can provide the basis of MA in this case.

As is the case for all medicinal products, information collected in the assessment of pre-clinical and clinical data finds its way into the Summary of Product Characteristics (SmPC). For epicutaneous PTs, this encompasses a number of valuable hapten-specific information, e.g., cross-reactions, which products should not be tested in children, which substances frequently lead to erythematous and/or irritant reactions and should therefore only be tested if there is a clear corresponding clinical suspicion, and which substances should be applied for only 24 h instead of 48 h. In addition, the SmPC informs the physician on products and product groups which can require a late reading between day 7 and 10 after PT-application. Also, hapten-specific information on the respective risk of active sensitization is provided, as well as information on other adverse effects, such as potential depigmentation of the skin or the risk of persistent test reactions.

## 4. Why Use Authorized Products?

As outlined above, the MA process as well as the maintenance post-authorization in line with, e.g., the variation regulation (EC 1234/2008) [45] ensures persistent quality, safety and efficacy of medicinal products. As described, the SmPC accompanying authorized epicutaneous PT preparations offers valuable general as well as hapten-specific information to the physicians, ensuring safe and efficacious use of the respective product. Although differences in quality between authorized and non-authorized products might not be directly apparent to physicians, numerous potential deficiencies are accounted for by respective regulatory requirements (Table 3). Main potential clinical consequences are impaired reliability of diagnosis, including both false-positive and false-negative PT reactions, as well as adverse reactions like active sensitization.

Although the solitary potential quality deficiencies listed in Table 3 can be considered exceptional for a single epicutaneous PT preparation, several factors, including the variety of potential deficiencies, the number of haptens in the portfolio of the manufacturers, as well as the origin and nature of certain active substances, increase the probability of quality defects. The list emphasizes the importance of regulatory requirements as well as the importance of information collected as a consequence of these requirements, e.g., the shelf life assigned to a specific product based on guideline-conform stability studies using validated analytical methods. This should be kept in mind, especially in cases where the literature advises otherwise. For example, Joy et al. published a literature analysis of information on active substance content, implying that the actual stability of certain allergen preparations is longer than the assigned shelf life and that this information could help in allergen supply management in clinical practice [46]. We advise against such practices, given that the shelf life assigned to PT preparations in MA procedures is based on ICH-compliant stability studies on a representative number of batches. As outlined above, potential quality defects, e.g., resulting from longer storage, are highly difficult to detect in clinical practice. They should thus be avoided by using PT preparations only within the assigned shelf life.

## 5. Conclusions

Epicutaneous PT preparations are currently the only sufficiently reliable diagnostic tools for the identification of delayed-type sensitization in patients with suspected allergic contact dermatitis. However, assessment of the relevance of patch test results is usually confined to matching of test readouts with the patient’s medical history, exposure assessment and labelling of accused products, as for most haptens, no alternative/confirmatory test method is available. Lacking a validated positive control, potential quality deficiencies of PT preparations resulting in, e.g., false-negative or false-positive test results are almost impossible to discover in daily practice and are underreported in the literature. It is thus highly important that clinicians are aware of the potential consequences of using unauthorized PT preparations, especially regarding the reliability of their diagnosis, subsequent decision and impact on their patients.

The various general and product-specific challenges and potential quality defects that have been summarized in this review underline the highly meaningful process of marketing authorization and independent quality control for this class of medicinal products. As compiled in Table 3, adherence to regulatory requirements can counter a broad range of potential quality deficiencies in PT preparations. Therefore, it is advantageous to use authorized PT preparations whenever possible, as has been recommended [36].

## Figures and Tables

**Table 1:** Overview of types of marketing authorization applications in the EU.

Type of Application	Legal Basis	Description
Full marketing authorization	Article 8(3) of Directive 2001/83/EC	Based on the full data set, including product-specific data from clinical studies
Mixed marketing authorization	Article 8(3) in combination with Annex I Part II Section 7 of Directive 2001/83/EC	Based on a combination of reports from limited non-clinical and/or clinical studies and bibliographical references
Well-established use	Article 10a of Directive 2001/83/EC	Based on appropriate scientific literature, if the active substances have been in well-established medicinal use within the EU for at least 10 years and if efficacy and safety are evident

**Table 2:** Possible simplification of regulatory requirements for patch test preparations.

Standard Regulatory Requirements for *In Vivo* Diagnostics	Adapted Requirements for PT preparations
***Quality***	
Validation of the manufacturing process for each product	Option of matrix approach for process validation in case of an identical production process
GMP requirements must be fulfilled throughout the manufacturing process	Waiver of GMP requirements for active substance production at the supplier
Details on the manufacturing of the active substance need to be presented in the product dossier	Absence of information on active substance manufacturing is acceptable
A full set of stability data (in accordance with ICH requirements) needs to be submitted for MA	Commitments for stability studies may be acceptable when long-term stability data for at least one batch are available
***Non-clinic***	
Collection of non-clinical data in corresponding animal studies	Option of compilation of already existing data from the literature
***Clinic***	
Performance of Phase II dose-finding studies necessary to select the appropriate concentration	Data from expert associations and/or suitable literature can support the selection of an appropriate concentration
Phase III confirmatory efficacy trials necessary	Relevant data from registries may be acceptable
Determination of sensitivity and specificity	Option to use alternative parameters like reaction index and positivity ratio

**Table 3:** Potential quality deficiencies (resulting in impaired diagnosis and/or adverse reactions) and regulatory countermeasures in authorized products.

Potential Quality Deficiencies	Resulting Potential Clinical Consequences (Impaired Reliability of Diagnosis and/or Safety)	Regulatory Countermeasures (Requirements for Each Hapten (no Extrapolation Possible):
**Manufacturing errors**		
Wrong active substance, e.g., due to a mix-up during production or a mistake by the supplier	- test result invalid, leading to a false diagnosis *Depending on the nature of the wrong active substance:* - risk of active sensitization or other adverse reactions	- active substance identity control using validated methods in the finished product
Suboptimal concentration of active substance, e.g., due to weighing error, or a missing definition of active substance shelf-life or general absence of content control by manufacturers	*Depending on the extent of deviation from the labelled/recommended active substance concentration:* - risk of false-negative results - risk of excessive positive reactions - risk of irritant or other adverse reactions - risk of active sensitization	- routine control of the manufacturing process - control of active substance content using validated methods both at release and in stability studies - definition of active substance shelf-life prior to and after initial opening of container
Inhomogeneity e.g., due to insufficient stirring during production	*Depending on the extent of inhomogeneity and thus deviation from the labelled/recommended active substance concentration in the applied dose:* - risk of false-negative results - risk of excessive positive reactions - risk of irritant or other adverse reactions	- control of homogeneity at release and in guideline-conforming stability studies in a validated method - validation of the manufacturing process, at least for a set of representative products
**Hapten-specific problems**		
Degradation or evaporation of active substance, e.g., during *manufacturing and/or storage*	*Depending on the extent of deviation from the labelled/recommended active substance concentration:* - risk of false-negative results - risk of irritant or other adverse reactions due to potentially harmful degradation products	- routine control of the manufacturing process - shelf-life determined based on stability data generated using validated methods in guideline-conform stability studies - photostability studies if the product is not stored protected from light
Reactions between the hapten and the container	*Depending on the nature and extent of interaction:* - risk of false-negative results - risk of false-positive results - irritant reactions	- assessment of the suitability of the container closure system - control of patch test quality in guideline-conform stability studies
Particle growth or formation, e.g., during *manufacturing and/or storage*	*Depending on particle size/hapten:* - sandpaper effect - potentially reduced bioavailability of the active substance - irritant reactions	- control of particle size at release and in guideline-conform stability studies in a validated method
**Contaminations**		
Contamination with impurities, e.g., due *to* the *supplier’s manufacturing process or source material*	*Depending on the nature of impurity:* - risk of false-positive results - risk of irritant reactions	- active substance content control in validated methods in the finished product - information on impurities to be provided for MAA - control of critical impurities by patch test manufacturer (if not controlled by supplier)
Contamination with other haptens, e.g., due to insufficient line clearance/cleaning regime	- risk of false-positive results - risk of active sensitization or other adverse reactions	- active substance content control in validated methods in the finished product - GMP requirements are effective upon active substance arrival at the PT manufacturer
Microbiological contamination, e.g., in the case of natural substances	- in case of, e.g., irritant patch test reaction, risk of infection - risk of false-positive reactions	- control of microbiological quality at finished product release and in stability studies using Ph. Eur. methods
**Product life-cycle**		
Change in critical active substance quality parameters due to a change in the supplier	*Depending on the extent of difference between active substances:* - no reproducibility of test results - risk of false-negative results - risk of excessive positive reactions - risk of irritant and other adverse reactions	- assessment of comparability pre/post change in active substance supplier and finished product batch-to-batch consistency in the mandatory variation procedure

## Abbreviations

The following abbreviations are used in this manuscript:

2-HEMA	2-hydroxyethyl methacrylate
DCP	decentralized procedure
DKG	German Contact Dermatitis Research Group
EU	European Union
GMP	good manufacturing practice
ICH	International Council for Harmonisation of Technical Requirements for Pharmaceuticals for Human Use
LTT	lymphocyte transformation test
MA	marketing authorization
MAA	marketing authorization application
MBT	mercaptobenzothiazole
MDBGN	methyldibromoglutaronitrile
MMA	methyl methacrylate
MRP	mutual recognition procedure
MS	member state
Ph. Eur.	European Pharmacopoeia
PR	positivity ratio
PT	patch test
PTD	p-toluene diamine
RI	reaction index
SmPC	summary of product characteristics
